# Supplementary material for: Transformation of financial institutions grants from the government to inclusive financial institutions in Indonesia
Source: PLoS One. 2023 Jun 23;18(6):e0286482. doi: 10.1371/journal.pone.0286482 (PMC10289368; doi:10.1371/journal.pone.0286482)
Supplement: Attachment — Submitted filename: author respond to reviewers comments-7mar23.docx [file pone.0286482.s003.docx]

Comment from reviewer and Author Respond

| No | Comment from reviewer | Author Respond |
| --- | --- | --- |
|  | **Reviewer 1** |  |
|  | The topic is very interesting. The authors have done a good job for determining the transformation of MFI from government grants to inclusive financial institutions in Indonesia that can help the funding needs of people’s economic businesses in rural areas. This paper provides some important insights for practitioners in Indonesia and countries that aim to reduce poverty and create job opportunities in villages. I appreciate the opportunity to review the proposed article and hope my considerations will help improve the work. |  |
| 1 | The abstract section has clearly mentioned the research objectives. However, adding the innovation and contributions, such as the differences between other literatures, is recommended. | An explanation of innovations and contributions to research compared to other publications has been included in the abstract section, (lines 17-19) and the use of MDS analysis (lines 23-26). |
| 2 | Please add line and page numbers so that pinpointed changes can be recommended. | Page numbers and line numbers have been added to the paper (see the manuscript) |
| 3 | There are some words for introducing MFI in section of results and discussions, I think it may be easier to read if make these sentences as another section after the introduction part. | The explanatory paper on MFIs has been covered in the methodology on terminology definitions. We also add some words that quite often we use in the sentence to make it easier for readers (lines 212-231). |
| 4 | This paper wrote in the methodology section “The study used the cluster proportional sampling technique. We employ the technique because this is follow-up research carried out in 2018, 2019 and 2022”. It is recommended add the relevance and importance of this method to justify the application in this study or explain clearly why the reason for using this technique is the follow-up research carried out in 2018, 2019 and 2022. | The relevance and importance of methodologies utilized in line with the research aim have been detailed in depth in the methodology section (lines 147-187; 203-210). |
| 5 | This paper used the cluster proportional sampling technique and analyzed the data well. However, the study aims to determine the transformation of MFI from government grants to inclusive financial institutions in Indonesia that can help the funding needs of people’s economic businesses in rural areas. I think this method can show the transformation but cannot show the relevance with PUAP activities. And this method cannot show the impact on funding needs of people’s economic business in rural areas either. Thus, other methods, such as OLS and event study, should also be used in this research. | Using cluster proportional sampling procedures, qualitative descriptive analysis techniques, and Multidimensional Scaling (MDS) analysis is sufficient in line with the study aims, limits, and research setting. The OLS approach is not yet necessary in this research. |
| 6 | It is recommended to add limitations and future research ideas in the conclusion section. And the implications for Indonesia from the results should also be added. | The findings concerning the limits and future research suggestions have been provided. Furthermore, the consequences of research findings for Indonesia have been included in conclusion (lines 552-556). |
|  | **Reviewer 2** |  |
|  | This manuscript investigates what has been made in the context of the transformation of financial institutions grants from the government to the inclusive financial institution and whether the microfinance institution is sustainable. The complete data, the comprehensive presentation, and the systematic storytelling are my first impressions of this manuscript. Using Indonesia as the object of study and the full elaboration of its figurative meanings are also strengths of the manuscript. Together with the findings, the manuscript’s discussion forms a clear marginal contribution to the existing literature. However, I think the research design may be inaccurate. Specifically, the manuscript has the following issues: |  |
| 1 | First, the manuscript is too close to the form of a research report. I recommended that the study’s intent be supplemented with a complete description of the manuscript’s structure. A more detailed description of the remainder of the manuscript can be added at the end of the Introduction. Section/Subsection that is not directly related to the central strand of research can be placed in the appendices if necessary. | The paper has been revised based on the reviewer's suggestions, and we tried to make it suit the standard for publication in the journal PLOS ONE (see the manuscript) |
| 2 | Second, the manuscript takes Indonesia as the default context but does not sufficiently argue in the background statements in the Introduction section whether Indonesia-specific issues are enough general or representative, despite their undoubted importance. In particular, the manuscript’s assessment of findings and policy implications needs to be more widespread; that is to say, although we use Indonesia as a sample, we would like to obtain a more general summarization, both from the perspectives of results and policy. Solving the first issue helps to improve the present issue. | We conducted the study in response to concerns about PUAP's numerous failures as a government initiative. Hundreds of Gapoktans failed to create MFIs for unknown reasons. This study focused on five successful MFIs changing into autonomous, independent financial institutions. The findings of this study can be utilized to inspire government strategies to make PUAP implementation more successful. We also have added cases in other countries that face almost similar obstacles to Indonesia in the 'introduction section' (lines 48-54). |
|  | Third, when presenting the research’s background and arguing its importance, it is recommended to add a general picture of such a subject. For example, plot a general state of the country or the world in Figure 1 as a comparison. As far as I can see, after these elements with a broader perspective are added to the research background statement, the importance and significance of the consequential research contents may become more apparent. | In the introduction, we discussed the issues that farmers in Indonesia and other countries face due to the condition of agriculture and the limited cash available to them. This work can be generalized to other countries for future research (see the manuscript) (lines 48-54). |
